# Electrospun Poly L-Lactic Acid Nanofiber Webs Presenting Enhanced Piezoelectric Properties

**DOI:** 10.3390/polym16030347

**Published:** 2024-01-28

**Authors:** Seung Kwan Hong, Jae-Jin Lee, Kap Jin Kim, Suk-Won Choi

**Affiliations:** 1 Department of Advanced Materials Engineering for Information & Electronics, Kyung Hee University, Yongin 17104, Republic of Korea; hsk93@khu.ac.kr (S.K.H.); kjkim@khu.ac.kr (K.J.K.); 2 Integrated Education Institute for Frontier Science & Technology (BK21 Four), Kyung Hee University, Yongin 17104, Republic of Korea

**Keywords:** piezoelectric, sensors, nanoparticles

## Abstract

There has been extensive research on electrospun ferroelectric nanoparticle-doped poly L-lactic acid (PLA) nanofiber web piezoelectric devices. In this study, BaTiO_3_ nanoparticles (BTNPs) were incorporated into the PLA to enhance the piezoelectric properties. The composite nanofiber webs were characterized using field emission scanning electron microscopy, energy-dispersive X-ray spectroscopy, and X-ray diffraction. The piezoelectric behavior was analyzed by measuring the peak-to-peak output voltage (V_p-p_) of the samples. The sensors fabricated from the PLA/BTNP nanofiber webs exhibited higher V_p-p_ values than the conventional electrospun PLA sensors. Furthermore, the corona-poled PLA/BTNP nanofiber web sensors exhibited even higher V_p-p_ values than the non-corona-poled sensors. Lastly, the effect of stacking nanofiber webs in terms of enhancing the sensor performance was also evaluated.

## 1. Introduction

The increasing adoption of nanofiber technology in sensor applications has facilitated the demand for nanofiber webs with enhanced performance capabilities [1]. Commercial pressure sensors can be categorized into piezoelectric, piezoresistive, and piezocapacitive sensors [2]. Poly L-lactic acid (PLA) is an eco-friendly polymer that possesses high biodegradability and biocompatibility. It exhibits piezoelectric properties due to its unique chiral conformation [3,4]. Consequently, PLA has been implemented as an eco-friendly piezoelectric material for various piezoelectric sensor applications. However, its piezoelectric properties must be further improved for more sensitive sensors.

Several studies have analyzed the piezoelectric behavior of PLA films, and their behavior has been compared to that of polyvinylidene fluoride (PVDF) films [5]. PVDF films require poling to induce piezoelectric behavior by aligning the C–F dipoles with the poling direction. A uniaxially drawn PLA film exhibits no remnant polarization; however, the surface charge of the PLA film is developed using shear stress, resulting in a shear piezoelectric signal without poling treatment. Our recent study revealed that unlike uniaxially drawn PLA films, PLA nanofiber webs accumulate surface charges under normal stress when exposed to the shear stress generated during the electrospinning process. This may be attributed to the remnant polarization caused by the very high DC electric field generated during electrospinning [6], which is similar to PVDF and polyvinylidene fluoride trifluoroethylene (PVDF/TrFE) with poling treatment.

Recent studies have demonstrated that the incorporation of nanorods or particles, such as ZnO [7], InN [8], and GaN [9], into PVDF or PVDF/TrFE can improve their piezoelectric characteristics. There has been a considerable rise in the number of studies focusing on piezoelectric PLA materials, and integrating nanorods or particles has also garnered significant interest [10,11,12]. BaTiO_3_ nanoparticles (BTNPs) are ferroelectric material with a high piezoelectric strain constant (d_33_ = 300–400 pC/N), and enhanced remnant polarization is exhibited after electrospinning a binary composite of PLA and BTNPs. In this study, we developed electrospun PLA/BTNP composite nanofibers, which exhibit highly improved piezoelectricity in response to normal stress compared to electrospun PLA nanofiber webs without BTNPs. The fabricated PLA/BTNP composite nanofibers were characterized using differential scanning calorimetry (DSC), field emission scanning electron microscopy (FE-SEM), energy-dispersive X-ray spectroscopy (EDX), and X-ray diffractometry (XRD). The piezoelectric characteristics of the fabricated sensors were monitored in the normal stress mode with the remnant polarization of the C=O dipole moment. Furthermore, the sensors fabricated from electrospun BTNP-doped PLA nanofiber webs using the corona-poling treatment exhibited significantly enhanced piezoelectric characteristics when compared to those fabricated from the electrospun PLA nanofiber webs without corona-poling treatment. Lastly, the effect of stacking the nanofiber webs was also evaluated.

## 2. Experimental

### 2.1. Preparation of the Electrospun PLA/BTNP Nanofiber Webs

Poly L-lactic acid (PLA) with a molecular weight (Mw) of 120,000 Da (4032D) was provided by NatureWorks (Minneapolis, MN, USA). Initially, the PLA was dissolved in chloroform (CF) at room temperature and stirred continuously. Dimethylformamide (DMF) was then added to create the final solution with a concentration of 9 wt. % for electrospinning. The CF:DMF ratio was 3:1. The PLA solutions doped with BTNPs were prepared by adding 5 parts per hundred resin (phr), 10 phr, and 15 phr of BTNPs simultaneously. Each solution was stirred using a magnetic stirrer at 30 °C for 12 h and later sonicated for 90 min at 30 °C. A magnetic stirrer is a widely used laboratory device that creates a rotating magnetic field. This device is used to rapidly spin a stir bar immersed in a solvent, facilitating the stirring or mixing of a solution. The resulting homogeneous white solutions were used for electrospinning.

**Figure 1:**
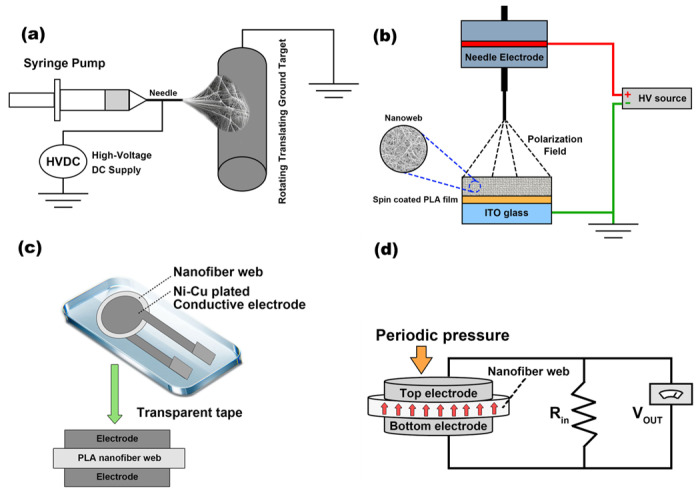
(**a**) Electrospinning experimental setup. (**b**) Corona-poling setup. (**c**) Sensor structure. (**d**) Custom-built dynamic pressure instrument.

The nanofiber webs were electrospun using a horizontal electrospinning experimental setup (as shown in Figure 1a). Initially, 12 mL of each PLA/BTNP solution was drawn into a syringe and electrospun under the following conditions. Needle type: 18 G; flow rate: 1.6 mL/h; supplied voltage: 12 kV; tip-to-collector distance: 10 cm; collector rotation rate: 60 rpm. Consequently, the electrospun PLA nanofiber webs and PLA/BTNP composite nanofiber webs were obtained, each with a thickness of 80 ± 2 µm.

### 2.2. Corona-Poling Process

Figure 1b presents a schematic diagram of the corona-poling setup used to increase the remnant polarization in the nanofiber webs [13]. Here, indium tin oxide (ITO) glass (2 × 2 cm) was spin-coated with a 1 μm PLA film. When the film was dry, nanofiber webs were electrospun onto the PLA film. A ground wire was then attached to the ITO glass, and a DC voltage of 10 kV was applied to the needle to produce an electric field; the treatment was performed for 10 min.

### 2.3. Fabrication of the Piezoelectric Sensors

The piezoelectric sensors were fabricated using BTNP-doped and corona-poled electrospun PLA nanofiber webs. The nanofiber webs were sandwiched between two circular pieces of nickel-copper-plated polyester fabric, which were adhered to both surfaces of the nanofiber web using a conductive adhesive, as shown in Figure 1c. Lastly, the entire structure was encapsulated using transparent adhesive tape.

### 2.4. Piezoelectricity Measurement

The output peak-to-peak voltage (V_p-p_) was measured using a custom-built dynamic pressure instrument (Figure 1d). To generate a piezoelectric signal, 1–5 kgf of normal pressure was applied to the sensor in a 10 Hz square wave with a 50% duty cycle. The piezoelectric signal generated by the periodic external pressure on the sensor was transferred to a Piezo film lab amplifier (TE Connectivity, Berwyn, PA, USA) set to the voltage mode with an R_in_ of 1 GΩ and a gain of 0 dB. Lastly, a noise-filtered output signal was obtained by passing the output signal through a passive band-pass filter with cut-off frequencies of 0.1 Hz for high-pass filtering and 10 Hz for low-pass filtering. The output signals were saved on a desktop computer using a NI-DAQ board (National Instruments, Austin, TX, USA). It is preferable to evaluate the piezoelectric coefficient of the PLA/BTNP composite nanowebs rather than assessing the V_p-p_. This preference may be attributed to the fact that the V_p-p_ may depend on the dimensions of the device. In this study, the V_p-p_ was meticulously evaluated in devices of identical dimensions. Consequently, the differences in device dimensions may have had a minimal impact on the piezoelectric properties in this evaluation.

### 2.5. Characterization

FE-SEM was used to characterize the microstructure and morphology of the nanofibers (SUPRA 55; Carl Zeiss Inc., White Plains, NY, USA). All the samples were coated with gold. EDX was performed on similar samples to detect the doping of the BTNPs into the nanofiber webs. The crystalline structure of the nanofibers was measured using an XRD equipped with a general area detector diffraction system (GADDS, D8 DISCOVER, Bruker AXS GmbH, Karlsruhe, Germany, Cu Kα radiation, λ = 0.154 nm) in the transmission mode. The X-ray scans were performed over a 2θ range of 3°–90°. Attenuated total reflectance infrared spectroscopy (ATR-IR) was performed using a diamond crystal accessory (GladiATR^TM^, PIKE Technologies, Fitchburg, WI, USA) attached to a Fourier transform infrared spectrophotometer (IFS 66v, Bruker, Germany) at a resolution of 4 cm^−1^ with 100 scans. The thermal characteristics of the PLA nanofiber webs, including the melting temperature (T_m_) and melting enthalpy (ΔH_m_), were analyzed using DSC (Diamond DSC, PerkinElmer, Shelton, CT, USA) at a heating rate of 20 °C/min.

## 3. Results and Discussion

### 3.1. PLA/BTNP Nanofibers

**Figure 2:**
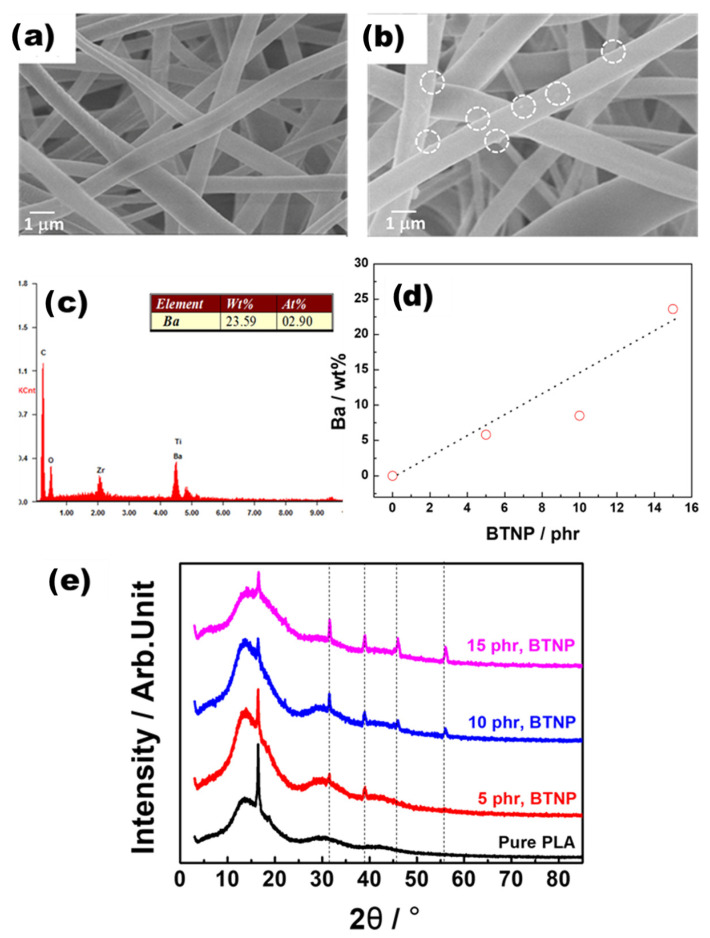
(**a**) FE-SEM micrograph of the pure electrospun PLA nanofiber web. (**b**) FE-SEM micrograph of the electrospun PLA nanofiber web with 5 phr BTNPs. (**c**) Typical EDX spectra of the electrospun PLA nanofiber webs with 15 phr BTNPs. (**d**) Average Ba elements of PLA nanowebs with different BTNP contents. (**e**) XRD profiles of the prepared samples.

We observed that the BTNPs were effectively introduced into the electrospun BTNP-doped PLA nanofibers. Figure 2a depicts the FE-SEM micrograph of the PLA nanofiber web, which shows a clear surface without any particles. Here, the nanofiber diameter lies within the range of 650–750 nm. Figure 2b depicts the FE-SEM micrograph of the PLA nanofiber web with 5 phr BTNPs, which contains some unknown particles on the nanofiber surfaces. EDX was performed to elucidate the unknown particles. Figure 2c,d depict the EDX spectra of the electrospun PLA nanofiber webs with 15 phr BTNPs and the average weight percentage (wt%) of barium (Ba) elements in the PLA nanowebs with varying BTNP contents, respectively. The increase in the BTNP content was reflected in the heightened peaks associated with Ba and titanium [14]. As the BTNP contents increased, there tended to be a linear increase in the average wt% of the Ba elements in the PLA nanoweb. Figure 2e depicts the typical XRD profiles of the prepared samples. As the BTNP content increased, the intensity of the typical XRD peaks [14] of BTNPs at 2θ = 32°, 39°, 46°, and 56° also increased. The FE-SEM, EDX, XRD, and DSC measurements demonstrated that the electrospun PLA/BTNP nanofiber webs were successfully fabricated.

### 3.2. Sensor Performances

**Figure 3:**
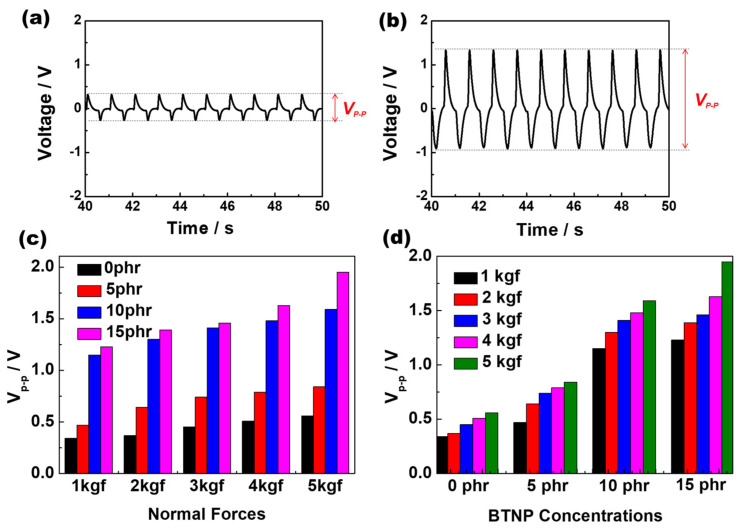
Typical profiles of piezoelectric voltages (V_p-p_) of the sensors fabricated from electrospun (**a**) PLA and (**b**) 15 phr BTNP−doped electrospun PLA nanofiber webs. (**c**) The dependence of V_p-p_ on BTNP content under different normal forces (pressure). (**d**) The dependence of V_p-p_ on normal forces under different BTNP concentrations.

Figure 3a,b depict the typical profiles of the piezoelectric voltages (V_p-p_) of the sensors fabricated from electrospun PLA and 15 phr BTNP-doped electrospun PLA nanofiber webs, respectively. It can be observed that the sensor with added BTNPs presented a larger V_p-p_ value than the pure PLA sensor. Figure 3c depicts the V_p-p_ against the BTNP content under different normal forces (pressure). As indicated in Figure 3c, the V_p-p_ exhibited a proportional relationship with the BTNP content from 0 to 10 phr. However, above 10 phr, the V_p-p_ tended to deviate from proportionality under the same pressure. This deviation may be attributed to the increased concentration of BTNPs, leading to agglomeration in the nanoweb and subsequently enhancing the stiffness of the composite web [11]. This, in turn, results in reduced piezoelectric deformation under the same pressure. Further, Figure 3d illustrates the dependence of V_p-p_ on normal forces at different BTNP concentrations. As depicted in Figure 3d, the V_p-p_ exhibited a tendency to proportionally increase in correlation with the rise in normal forces from 1 to 5 kgf under the same BTNP concentrations. The intrinsic PLA molecules have no remnant polarization; however, surface charge occurred in the PLA nanofibers due to the shear stress generated during the electrospinning process. This may be attributed to the change in the ratio of α-crystalline forms to β-crystalline forms. Essentially, the β-crystalline structures that tend to be dominant in the PLA molecules have undergone the electrospinning process. The C=O dipole groups that affect the residual polarization in the electrospun PLA molecules do not lie along the molecular chain direction but instead along the vertical direction, which is perpendicular to the molecular chain direction. Therefore, the electrospun PLA nanofiber webs exhibited remnant polarization on the surface, thereby increasing the piezoelectric voltage with a normal force applied to the sensors. 

**Figure 4:**
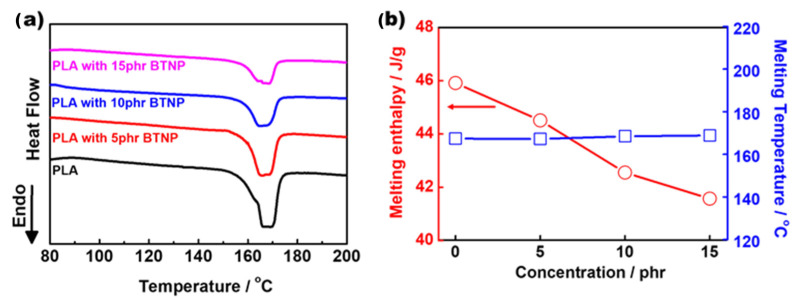
(**a**) DSC melting traces of electrospun PLA and electrospun PLA/BTNP nanofibers. (**b**) Melting enthalpies and temperatures of BTNP concentrations.

Furthermore, incorporating BTNPs into the electrospun PLA nanofibers can effectively improve the piezoelectric properties. Figure 4a depicts the DSC melting traces of the electrospun PLA and electrospun PLA/BTNP nanofibers. While the melting temperatures were relatively constant regardless of the BTNP content, the melting enthalpies exhibited a gradual decrease with the increase in the BTNP content, as shown in Figure 4b. The decrease in the melting enthalpies indicates a reduction in the degree of crystallinity of the PLA. This observation implies an increase in the β-crystalline form of the PLAs upon BTNP incorporation. The doping of the BTNPs, accelerating the β-crystalline conformation of the PLA, is crucial in enhancing the piezoelectric features since the increased amorphous regions in the BTNP-doped PLA can help in achieving higher ionic conductivity [15].

**Figure 5:**
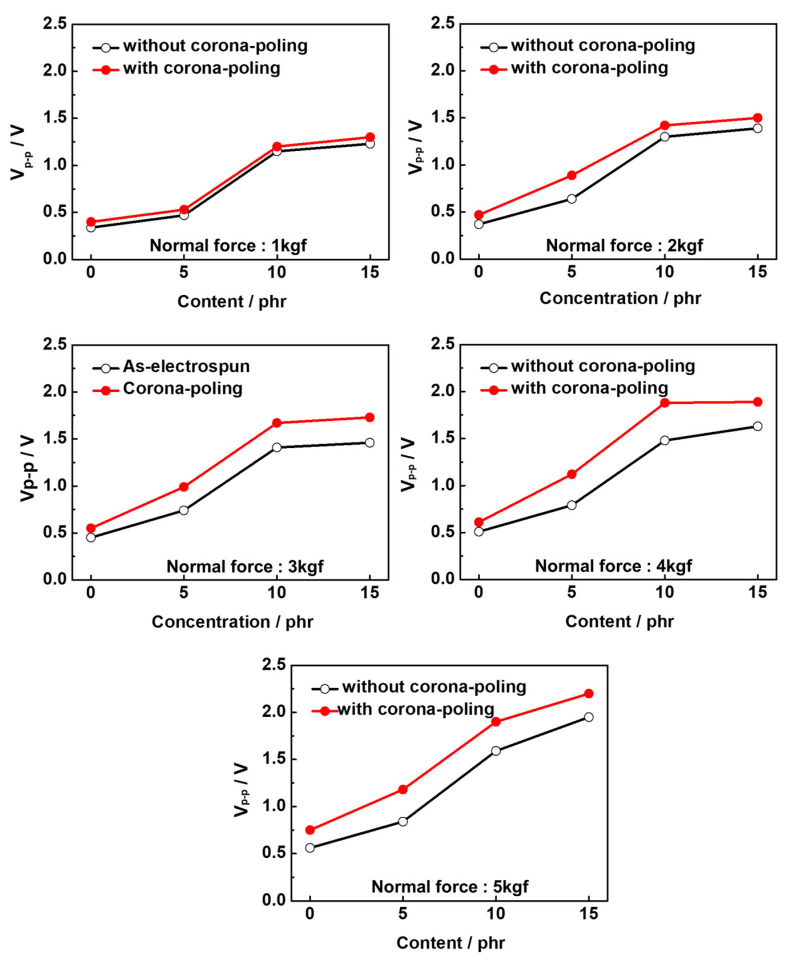
V_p-p_ values of sensors fabricated from PLA/BTNP nanofiber webs with and without corona-poling treatment as a function of the concentration of BTNPs and the normal force applied to the sensors.

To further enhance the piezoelectricity of the sensors, the nanofiber webs fabricated using electrospinning were subjected to a high-pressure corona-poling process. We compared the V_p-p_ values of the sensors fabricated from electrospun PLA/BTNP nanofiber webs with and without corona-poling treatment as a function of the concentration of BTNPs and the normal force applied to the sensors. The corona-poling treatment resulted in an increase in the V_p-p_ values of the sensors, as shown in Figure 5. The corona-poling process increases the remnant polarization of the PLA and BaTiO_3_, respectively, further enhancing their piezoelectric properties. Thus, corona-poling was confirmed to be an effective method for significantly enhancing the piezoelectricity of sensors fabricated from electrospun PLA/BTNP nanofiber webs. A detailed explanation of the mechanism of the corona-poling treatment enhancing the piezoelectric properties is given in the Supplementary Materials.

**Figure 6:**
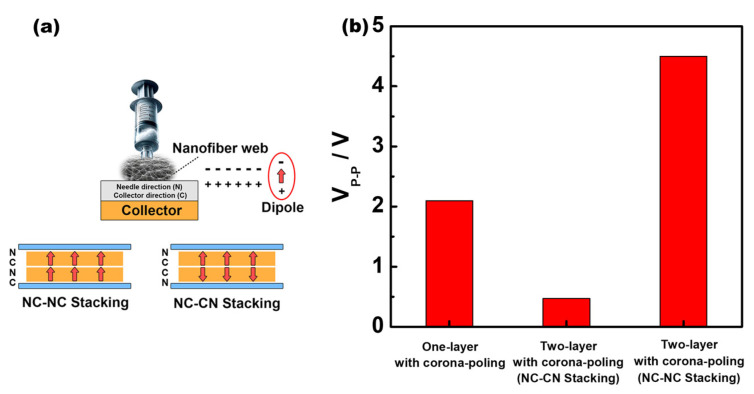
(**a**) Two kinds of sensors, such as with NC−NC and NC−CN stacking, wherein C and N are the collector and needle directions, respectively. (**b**) Comparison of V_p-p_ values of one-layer corona-poled 15 phr BTNP−doped PLA sensor, two-layer corona−poled 15 phr BTNP−doped PLA sensor with NC−CN stacking, and two−layer corona−poled 15 phr BTNP-doped PLA sensor with NC−NC stacking (normal pressure: 5 kgf).

Lastly, two types of sensors, such as with NC-NC and NC-CN stacking, were fabricated, wherein C and N represent the collector and needle directions, respectively. Figure 6a depicts the two types of piezoelectric sensors with NC-CN and NC-NC stacking configurations, which were prepared to analyze the effect of stacking the nanofiber webs. The V_p-p_ value of the one-layer corona-poled 15 phr BTNP-doped PLA sensor is 2.2 V, whereas that of the two-layer corona-poled 15 phr BTNP-doped PLA sensor with NC-CN stacking is 0.47 V (under 5 kgf), exhibiting a significant decrease, as shown in Figure 6b. This is because the NC-CN stacking configuration reduced the net dipole moment due to the C=O dipoles arranged in the opposite direction, thereby reducing the remnant polarization. Conversely, the V_p-p_ value of the two-layer corona-poled 15 phr BTNP-doped PLA sensor with NC-NC stacking is 4.5 V (under 5 kgf), which presents an approximately two-fold improvement. In this case, the NC-NC stacking configuration increased the net dipole moment owing to the C=O dipoles being arranged in the same direction, thereby increasing the remnant polarization. These results indicate that the net dipole moment caused by preferentially oriented C=O dipoles enhances the piezoelectric output voltage [16].

## 4. Conclusions

In this study, BTNP-doped PLA nanofiber webs were employed to enhance the piezoelectric properties of piezoelectric sensors. The uniform dispersion of BTNPs within the electrospun nanofiber webs was confirmed using FE-SEM, EDX, and XRD analyses. Additionally, we enhanced the piezoelectric behavior of the BTNP-doped PLA nanofibers by applying corona-poling to increase the remnant polarization. Furthermore, the piezoelectric characteristics of the BTNP-doped PLA nanofiber webs subjected to corona-poling treatment could be significantly improved by employing a two-layer stacking configuration with an NC-NC arrangement. This study elucidates that the preferential orientation of the C=O dipoles in the PLA nanofiber webs was facilitated by the stretching and poling effects during the electrospinning and corona-poling processes. The sensors comprising the electrospun PLA nanowebs with BTNP incorporation and corona-poling processes were more cost-effective and exhibited a superior piezoelectric performance compared to the sensors made from PVDF.

## Data Availability

The raw data supporting the conclusions of this article will be made available by the authors on request.

## Supplementary Materials

The following supporting information can be downloaded at https://www.mdpi.com/article/10.3390/polym16030347/s1, Figure S1: Piezoelectric characteristics of normal stress (T1) in the PLA/BTNP composite nanowebs after the corona-poling process.
