# Supplementary material for: Electrospun Poly L-Lactic Acid Nanofiber Webs Presenting Enhanced Piezoelectric Properties
Source: Polymers (Basel). 2024 Jan 28;16(3):347. doi: 10.3390/polym16030347 (PMC10857000; doi:10.3390/polym16030347)
Supplement: Supplementary file 1 [file polymers-16-00347-s001.zip › polymers-2783443-supplementary.pdf]

Supplementary Material

Significant differences were observed in the evaluated output voltages before and after corona poling. The mechanism of the corona-poling treatment to enhance the piezoelectric properties can be explained as follows:

Through the corona-poling process, C=O linkages in PLA webs were compelled to align with the direction of the surface normal, maximizing the $\boldsymbol{d_{11}}$, $\boldsymbol{d_{12}}$, $\boldsymbol{d_{13}}$, $\boldsymbol{d_{24}}$, and $\boldsymbol{d_{35}}$ associated with the remnant polarization of PLA/BTNP composite nanowebs, as shown in Figure S1. Consequently, the PLA/BTNP composite nanowebs, after the corona-poling process, may exhibit significantly enhanced piezoelectricity.

.

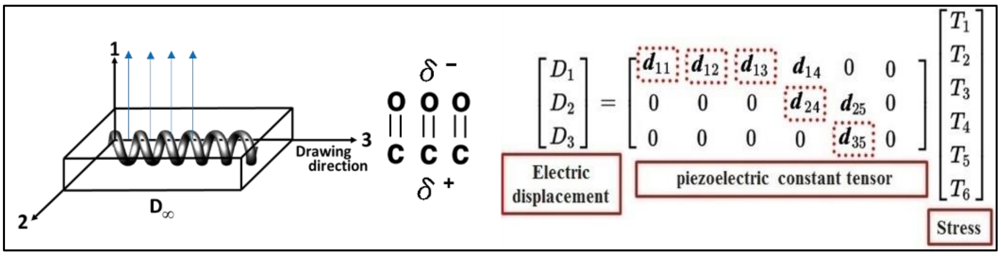

Figure S1. Piezoelectric characteristics of normal stress ($\boldsymbol{T_1}$) in the PLA/BTNP composite nanowebs after corona-poling process.

As shown in Fig. S1, the charge between the top and bottom electrodes on surface 1 during both normal and shear stress can be described as $\boldsymbol{D_1 = d_{11}T_1 + d_{12}T_2 + d_{13}T_3 + d_{14}T_4}$. When only the normal stress $\boldsymbol{T_1}$ is present, $\boldsymbol{D_1 = d_{11}T_1}$. Thus, the PLA/BTNP composite nanowebs, after the corona-poling process, should primarily respond to the normal stress $\boldsymbol{T_1}$.
